# A novel classification of testicular sex cord‐stromal tumours from the Testicular Sex Cord‐Stromal Tumour (TESST) group: a collaboration of the Genitourinary Pathology Society (GUPS) and the International Society of Urological Pathology (ISUP)

**DOI:** 10.1111/his.70207

**Published:** 2026-07-08

**Authors:** Andres M. Acosta, Felix Bremmer, John C. Cheville, Maurizio Colecchia, Eva Comperat, Kristine Cornejo, Dilek E. Baydar, Anthony J. Gill, Maria del Pilar Gonzalez‐Peramato, Sounak Gupta, Michelle S. Hirsch, Muhammad T. Idrees, Chia‐Sui Kao, Fiona MacLean, Andres Matoso, Rohit Mehra, Santosh Menon, Kvetoslava Michalova, Sambit K. Mohanty, Miguel Reyes‐Mugica, Maria Rosaria Raspollini, Ankur R. Sangoi, Lynette M. Sholl, Stephanie Siegmund, Satish K. Tickoo, Toyonori Tsuzuki, Thomas M. Ulbright, Sean R. Williamson, Asli Yilmaz, Daniel M. Berney

**Affiliations:** ^1^ Department of Pathology Indiana University Indianapolis IN USA; ^2^ Institute of Pathology University Medical Center Göttingen Göttingen Germany; ^3^ Department of Pathology Mayo Clinic Rochester MN USA; ^4^ Department of Pathology Universita Vita Salute San Raffaele Milan Italy; ^5^ Department of Pathology Medical University Vienna Vienna Austria; ^6^ Department of Pathology Koc University School of Medicine Istanbul Turkey; ^7^ Department of Pathology, Sydney Medical School University of Sydney Sydney NSW Australia; ^8^ Department of Pathology University Hospital La Paz, Universidad Autónoma de Madrid Madrid Spain; ^9^ Department of Pathology MassGeneralBrigham/Harvard Medical School Boston MA USA; ^10^ Department of Pathology and Laboratory Medicine, Diagnostics Institute Cleveland Clinic Cleveland OH USA; ^11^ Department of Pathology, Douglass Hanly Moir Pathology Sonic Healthcare Sydney NSW Australia; ^12^ Department of Pathology Johns Hopkins University Baltimore MD USA; ^13^ Department of Pathology University of Michigan Ann Arbor MI USA; ^14^ Department of Pathology Tata Memorial Centre Mumbai India; ^15^ Department of Pathology Charles University, Faculty of Medicine in Plzen, Biopticka Laborator, Ltd Plzen Czech Republic; ^16^ Department of Pathology CORE Diagnostics Gurgaon Haryana India; ^17^ Department of Pathology University of Miami Miller School of Medicine Miami FL USA; ^18^ Histopathology and Molecular Diagnostics University Hospital Careggi and University of Florence Florence Italy; ^19^ Department of Pathology Stanford University Stanford CA USA; ^20^ Department of Pathology Memorial Sloan‐Kettering Cancer Center New York NY USA; ^21^ Department of Surgical Pathology Aichi Medical University Nagakute Japan; ^22^ Department of Diagnostic and Molecular Pathology Alberta Precision Labs and University of Calgary Calgary AB Canada; ^23^ Centre of Cancer Biomarkers and Biotherapeutics, Barts Cancer Institute, Charterhouse Square Queen Mary University of London London UK

**Keywords:** classification, granulosa cell tumour, Leydig cell tumour, Sertoli cell tumour, sex cord‐stromal tumour, testis

## Abstract

**Aims:**

Testicular sex cord‐stromal tumours (TSCSTs) are rare neoplasms that arise from (or show differentiation to) elements derived from the sex cords or intertubular stroma. The current classification (World Health Organization, 2022) is based almost entirely on morphology, with many tumour types such as granulosa cell tumours being defined based on their resemblance to ovarian counterparts. Recent studies have uncovered new clinicopathological, molecular and biological nuances within this group of tumours, underscoring differences with ovarian homologues.

**Methods and results:**

Considering the new data, a group of genitourinary pathology experts (Testicular Sex Cord‐Stromal Tumour [TESST] group) has proposed a revised ‘TESST classification’ that incorporates the best available evidence. This classification is structured and hierarchical, composed of the following tiers: (1) group, (2) category, (3) entity and (4) histological pattern. ‘Group’ refers to the histological compartment a specific tumour belongs to (sex cords, stroma or both), whereas ‘category’ is defined by the phenotype of the tumour, taking non‐neoplastic elements of the ovary and testis as a reference. The third tier, ‘entity’, refers to neoplasms with shared clinicopathological and/or molecular features that justify classifying them as distinct types. Lastly, ‘histological patterns’ are recurrent morphological variations within entities that are not linked to distinct clinicopathological or molecular features.

**Conclusions:**

We propose a classification that will provide a framework to improve current clinical management and guide future research in the field.

## Graphical abstract

The TESST classification of testicular sex cord‐stromal tumours was agreed upon by consensus based on the best available evidence. The picture shows the members of the TESST group and delegates of GUPS and ISUP present in‐person at the first meeting of the group held at Johns Hopkins Hospital (Baltimore, 2024).

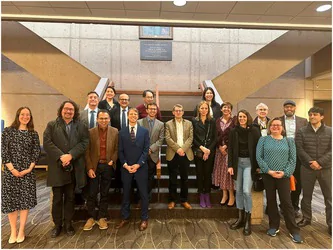

**Abbreviations**
GOF: Gain‐of‐function
GUPS: Genitourinary Pathology Society
ISUP: International Society of Urological Pathology
LOF: Loss‐of‐function
TSCSTs: Testicular sex cord‐stromal tumours

## Background

Testicular sex cord‐stromal tumours (TSCSTs) are relatively rare and often diagnostically challenging. Historically, they were grouped under one of four major categories: Leydig cell tumour, Sertoli cell tumour, granulosa cell tumour and unclassified sex cord‐stromal tumour. Over recent years, additional histological types have been introduced. However, the current classification still relies entirely on morphology, with a few tumour types being defined based on their resemblance to ovarian counterparts.^1^

With the advent of modern technologies, molecular analyses of TSCSTs have identified recurrent drivers and genomic alterations in several TSCST types, including some associated with aggressive clinical behaviour. Considering these new data, the Testicular Sex Cord‐Stromal Tumour (TESST) group has recently issued recommendations for workup and classification of specific TSCST types.^2^ However, we believe the current framework requires an overarching reappraisal to incorporate newly recognized tumour types and molecular data into a logical scheme that is flexible enough to accommodate future entities. To this end, we proposed a new structured classification of TSCSTs agreed upon by consensus and based on the best available evidence.

## Materials and Methods

This classification was proposed by the TESST group, which currently consists of 30 members from four continents (the Americas, Europe, Asia and Oceania). The group's activities were sponsored jointly by the Genitourinary Pathology Society (GUPS) and the International Society of Urological Pathology (ISUP).

This TESST classification is based, in part, on the recommendations for classification that resulted from multiple consensus activities held by the group during 2023–2024 (including an in‐person meeting in Baltimore in March 2024). Details of interobserver variability among experts were previously shown, and some initial consensus statements were published in *Histopathology*.^2^, ^3^ Additionally, this classification incorporates items that were subject to further discussions and consensus activities held between late 2024 and early 2025 (including an in‐person meeting in Boston in March 2025). Further details regarding the consensus methodology are available in the recent white paper published by the TESST group.^2^

A first preliminary draft of the classification was circulated via e‐mail among the members of the group during the last quarter of 2024 and the first quarter of 2025 with a request for comments/suggestions. A second draft incorporating selected suggestions was presented during the latest in‐person TESST meeting (Boston, March 2025). On this occasion, additional items were put to a vote, based largely on the members' comments regarding the preliminary classification distributed via e‐mail.

## TESST classification of sex cord‐stromal tumours

### General Structure

We propose a classification characterized by a hierarchical structure containing the following tiers: (1) group, (2) category, (3) entity and (4) histological pattern. The first tier, ‘group’, refers to the general compartment that the tumours belong to according to their phenotype, namely sex cord compartment, stromal compartment or both (sex cord‐stromal tumours). The second tier, ‘category’, is defined by the non‐neoplastic cell type that the tumours recapitulate, when applicable (e.g. Leydig cells, Sertoli cells, granulosa cells). The third tier, ‘entity’, refers to neoplasms with shared clinicopathological and/or molecular features that support their interpretation as distinct tumour types. Finally, the fourth category, ‘histological pattern’, refers to morphological and/or immunophenotypic variations within an entity. The proposed complete classification is summarized in detail in Table 1. In the following sections, we will present the most relevant changes within each group compared to the current classification (WHO 2022), providing a rationale for implementing such changes.

**Table 1:** TESST classification of testicular sex cord‐stromal tumours

**Classification groups, categories, entities and histological patterns**	**Definition**
GROUP: SEX CORD TUMOURS	Testicular neoplasms showing differentiation to non‐neoplastic cell types derived from the sex cords
**Category: Sertoli cell tumours**	Sex cord tumour with Sertoli cell‐like phenotype
*Entities*:	
*Sertoli cell tumour, usual type*	Sex cord tumour with Sertoli cell phenotype lacking large cell morphology and a very strong association with GOF *CTNNB1* variants (>90% in primary tumours)
Histological patterns	
Sclerosing Sertoli cell tumour, usual type	Sertoli cell tumour, no specific subtype with abundant hyalinized collagenous stroma
Signet ring cell Sertoli cell tumour, usual type	Sertoli cell tumour, no specific subtype with a predominant population of univacuolated signet ring cell‐like cells
*Large cell calcifying Sertoli cell tumour (including pure intratubular tumours)* *	Sex cord tumour with large Sertoli cell‐phenotype, mulberry‐like calcifications, myxoid stroma, neutrophilic infiltrates showing and a very strong association with LOF *PRKAR1A* alterations (>90%)
*Large cell hyalinizing Sertoli cell neoplasia (including pure intratubular tumours)* *	Sex cord tumour with large Sertoli cell phenotype, largely (albeit not exclusively) intratubular growth, prominent deposits of basement membrane‐like material showing, and an almost invariable association with Peutz–Jeghers syndrome
**Category: Granulosa cell tumours**	Sex cord tumour with granulosa cell‐like phenotype
*Entities*:	
*Adult granulosa cell tumour*	Sex cord tumour with close morphological resemblance to ovarian adult granulosa cell tumour; *FOXL2* p.C134W is supportive (but not required)
*Benign juvenile granulosa cell tumour*	Benign, largely infantile, testicular sex cord tumour with close morphological resemblance to ovarian juvenile granulosa cell tumour
**Category: Other sex cord tumours**	Pure sex cord neoplasms other than Sertoli and granulosa cell tumour
*Entities*:	
*Inflammatory and nested testicular sex cord tumour*	Aggressive sex cord neoplasm with morphological evidence of sex cord differentiation (nested architecture), expression of EMA and CD30, and a strong association with *ATF1::EWSR1* or *EWSR1* rearrangement
*Sex cord tumour, no specific type*	Pure sex cord neoplasm that cannot be classified into one of the categories described above
GROUP: STROMAL TUMOURS	Testicular neoplasms with differentiation to non‐neoplastic cell types of the gonadal stroma
**Category: Leydig cell tumours**	Stromal tumour with Leydig cell‐like phenotype
*Entities*:	
*Leydig cell tumour, usual type*	Tumour with Leydig cell phenotype and no evidence of loss of FH function
*Leydig cell tumour, fumarate hydratase*‐*deficient*	Tumour with Leydig cell phenotype and evidence of FH‐deficiency
**Category: Other stromal tumours**	Pure stromal neoplasms other than Leydig cell tumour
*Entities*:	
*Spindle cell gonadal stromal tumour*	Pure spindle cell testicular neoplasm containing cells that resemble those of the intertubular stroma and/or peritubular myoid cells
Histological patterns	
Myoid gonadal stromal tumour	Pure spindle cell testicular stromal tumour showing co‐expression of SMA and S100
Fibroma/thecoma	Pure spindle cell testicular stromal tumour resembling ovarian fibroma/thecoma
*Stromal tumour, no specific type*	Pure stromal tumour that cannot be classified into the categories described above
GROUP: SEX CORD‐STROMAL TUMOURS	Testicular neoplasms containing a mixture of components with distinct sex cord and stromal phenotype
**Category: Mixed sex cord‐stromal tumours**	Testicular neoplasms containing a mixture of components with distinct sex cord and stromal phenotype
*Entities*:	
*Sertoli‐Leydig cell tumour*	Testicular neoplasm containing distinct components with Sertoli and Leydig cell phenotype, resembling ovarian counterparts; demonstration of DICER1 alterations is supportive (but not required)
*Mixed sex cord‐stromal tumour, no specific type*	Testicular neoplasm containing components with morphological evidence of sex cord differentiation intermingled with neoplastic stromal spindle cells arranged in fascicles or whorls; these tumours may contain germ cells (historically interpreted as an entrapped non‐neoplastic component)
Histological patterns	
Sertoli‐stromal cell tumour	Testicular neoplasm containing components with morphological evidence of Sertoli cell differentiation (e.g. tubules) intermingled with neoplastic stromal spindle cells arranged in fascicles; these tumours may contain germ cells (historically interpreted as an entrapped non‐neoplastic component)

GOF, Gain‐of‐function; LOF, Loss‐of‐function; TESST, Testicular Sex Cord‐Stromal Tumour group.

* Please refer to the corresponding section in the article for recommendations on the recommended diagnostic terminology.

#### Sex cord tumours group

This group comprises neoplasms with pure sex cord phenotype. Specifically, it includes Sertoli cell tumours, granulosa cell tumours, inflammatory and nested testicular sex cord tumour, and sex cord tumours that cannot be further categorized (sex cord tumour, no specific type). The proposed classification of sex cord tumours is presented below.

## Group: sex cord tumours

Category: Sertoli cell tumours

Entities:

- Sertoli cell tumour, usual type
  Histological patterns:
    - Sclerosing Sertoli cell tumour, usual type
    - Signet ring Sertoli cell tumour, usual type
- Large cell calcifying Sertoli cell tumour (including pure intratubular tumours)
- Large cell hyalinizing Sertoli cell neoplasia (including pure intratubular tumours)

Category: Granulosa cell tumours

Entities:

- Adult granulosa cell tumour
- Benign juvenile granulosa cell tumour

Category: Other sex cord tumours

Entities:

- Inflammatory and nested testicular sex cord tumour
- Sex cord tumour, no specific type

The most relevant changes introduced in this classification (when compared to the current WHO classification) are highlighted below.

### Changes Within the Group of Sex Cord Tumours

1. *‘Sertoli cell tumour, not otherwise specified’ is replaced by ‘Sertoli cell tumour, usual type’.*

Rationale: The phrase ‘usual type’ clearly states that these tumours lack features diagnostic of specific histological types such as large cell calcifying Sertoli cell tumour (which is considered an entity herein). In our opinion, this terminology is clearer than ‘not otherwise specified’, which may be misinterpreted to imply that a workup to determine the specific type was not performed or not available.

2 *Sclerosing Sertoli cell tumour and signet ring Sertoli cell tumour (formerly ‘signet ring stromal tumor’) are considered histological patterns of Sertoli cell tumour, usual type.*

Rationale: The overall clinicopathological features of these tumours are similar to those of otherwise typical Sertoli cell tumour, usual type. Specifically, they are largely indolent and show evidence of Sertoli cell differentiation without a large cell phenotype.^4^, ^5^, ^6^ Importantly, foci with signet ring‐like cells are not uncommon in typical Sertoli cell tumour, usual type. Moreover, like Sertoli cell tumour, usual type, both the sclerosing and signet ring cell patterns harbour gain‐of‐function *CTNNB1* variants and show diffuse nuclear beta‐catenin expression.^7^, ^8^ Whether tumours that are negative for ‘sex cord‐stromal tumour markers’ and positive for neuroendocrine markers resembling solid pseudopapillary tumours of the pancreas represent a distinct entity or not remains an open question that requires further studies.^9^ Therefore, we do not recommend changing the current classification to regard this as a distinct entity at this time.

3 *‘Large cell calcifying Sertoli cell tumour’ is replaced by ‘large cell calcifying Sertoli cell tumour (including pure intratubular tumours)’.*

Rationale: This change addresses the existence of rare pure intratubular large cell calcifying Sertoli cell tumours, which may show significant overlap with neoplasms seen in patients with Peutz–Jeghers syndrome.^10^, ^11^ Demonstration of *PRKAR1A* variants, loss of PRKAR1A expression by immunohistochemistry or an association with the Carney complex is likely needed to make the distinction between intratubular large cell calcifying Sertoli cell tumour and intratubular large cell hyalinizing Sertoli cell neoplasia.^10^

Due to the cumbersome nature of this entity's name, we recommend diagnosing these as ‘large cell calcifying Sertoli cell tumour’ (for invasive cases) or ‘intratubular large cell calcifying Sertoli cell tumour’ (for pure intratubular cases). However, we believe that keeping the entity's name as stated above is important, to raise awareness about the existence of rare pure intratubular tumours that may need to be distinguished from ‘intratubular large cell hyalinizing Sertoli cell neoplasia’.

4 *‘Intratubular large cell hyalinizing Sertoli cell neoplasia’ is replaced by ‘large cell hyalinizing Sertoli cell neoplasia (including pure intratubular tumours)’.*

Rationale: This change addresses the existence of *STK11*‐altered neoplasms with invasive components which, in the testis, appear to occur invariably in the context of Peutz–Jeghers syndrome (i.e. unlike ovarian *STK11*‐altered sex cord tumours, which may be sporadic).^11^, ^12^, ^13^ We believe that the ‘large cell calcifying Sertoli cell tumours’ previously reported in patients with Peutz–Jeghers syndrome are most likely *STK11*‐altered tumours related to ‘intratubular large cell hyalinizing Sertoli cell neoplasia’. Loss of STK11 expression (and retained PRKAR1A expression) has been demonstrated in at least 1 such tumour.^12^

As discussed in point 3, we recognize that the name of this entity is too cumbersome to use as a pathological diagnosis. Therefore, we recommend diagnosing these as ‘large cell hyalinizing Sertoli cell neoplasia’ (for invasive cases) or ‘intratubular large cell hyalinizing Sertoli cell neoplasia’ (for pure intratubular cases). Nonetheless, we believe that keeping the entity's name as stated above is important to raise awareness about the existence of rare, invasive tumours that may need to be distinguished from ‘large cell calcifying Sertoli cell tumour’.

5 *‘Juvenile granulosa cell tumour’ is replaced by ‘benign juvenile granulosa cell tumour’.*

Rationale: Unlike ovarian juvenile granulosa cell tumour, those arising in the testis are largely infantile (>90% occur within the first year of life), hormonally inactive and have not been reported in adult patients.^14^, ^15^ The molecular alterations described in ovarian juvenile granulosa cell tumour have not been identified in testicular counterparts, which instead show monosomy 10 as a recurrent finding (~60%).^16^, ^17^, ^18^, ^19^ Most importantly, testicular juvenile granulosa cell tumours appear to be invariably indolent and therefore cured by surgical resection.^14^, ^15^ Given that a subset of juvenile granulosa cell tumours of the ovary, which are significantly more common, show malignant clinical behaviour, the current terminology used for testicular counterparts may lead to overtreatment and excessive diagnostic workups. Therefore, the addition of the descriptor ‘benign’ is intended to highlight their indolent nature and avoid inappropriately aggressive clinical management.

6 *‘Inflammatory and nested testicular sex cord tumour’ is introduced as a new entity.*

**Figure 1:**
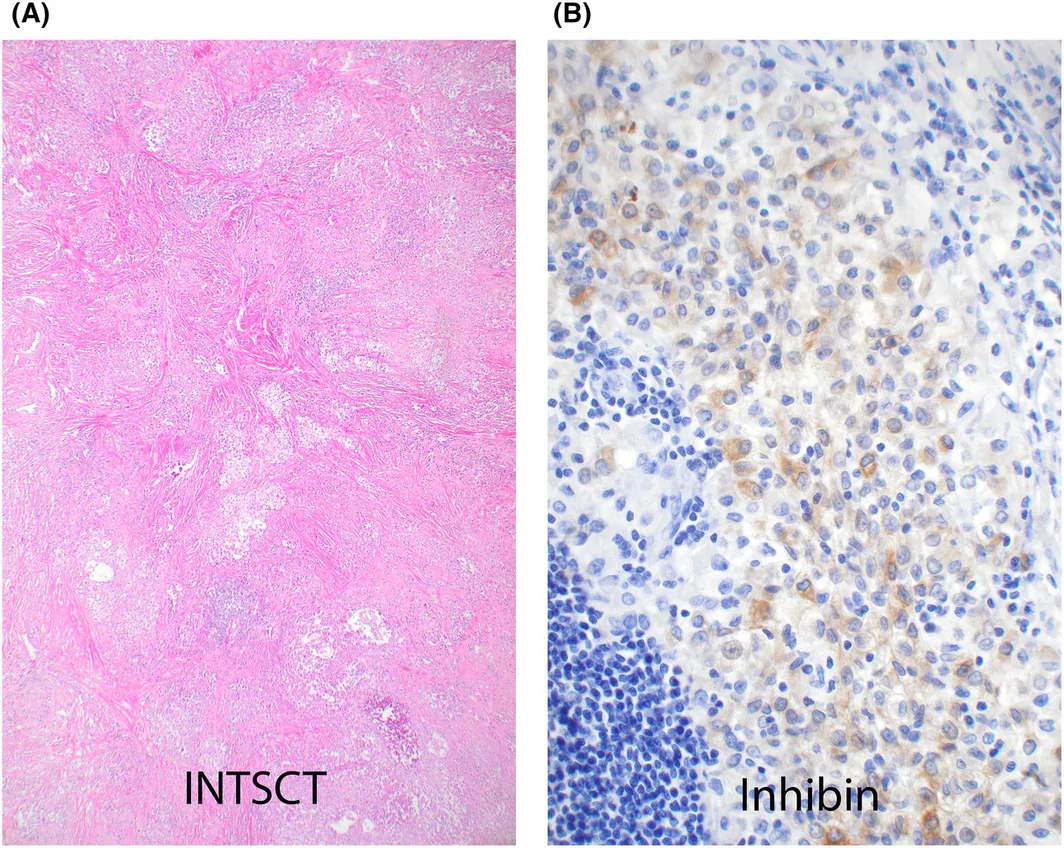
Inflammatory and nested testicular sex cord tumour (INTSCT, panel **A**). These tumours arise within the testicular parenchyma and show expression of sex cord‐stromal tumour markers such as SF1 and inhibin (inhibin is shown in panel **B**).

Rationale: Clinically malignant TSCSTs showing morphological resemblance to seminoma were first reported by Henley *et al*. in 2002.^20^ These tumours, which affected patients in a wide age range, exhibited a solid growth pattern consisting of variably sized nests and prominent mixed inflammatory infiltrates (Figure 1A,B). Many tumours compiled in this first series were originally misdiagnosed as seminoma, being subsequently reclassified as sex cord‐stromal tumours.^20^ Given that they show morphological and immunohistochemical evidence of sex cord differentiation, they were proposed to represent malignant examples of Sertoli cell tumour. However, these neoplasms show a somewhat distinct immunoprofile among sex cord‐stromal tumours in that they express EMA and CD30. Recently, molecular studies have demonstrated that they harbour recurrent *EWSR1::ATF1* fusions or *EWSR1* rearrangement.^21^, ^22^, ^23^ Their unique clinicopathological and molecular features support their interpretation as a distinct entity. Moreover, separating them from Sertoli cell tumours is clinically relevant, since the latter are largely benign (~90%), whereas inflammatory and nested testicular sex cord tumours should always be considered malignant/potentially malignant.

#### Stromal tumours group

Stromal tumours of the testis comprise a group of neoplasms that derive from (or show differentiation to) cells of the gonadal stroma. This group includes tumours with Leydig cell phenotype (the most common type of TSCST) as well as rare gonadal tumours which often show pure spindle cell morphology. The proposed classification of stromal tumours is presented below.

## Group: stromal tumours

Category: *Leydig cell tumours*

Entities:

- Leydig cell tumour, usual type
- Leydig cell tumour, fumarate hydratase‐deficient

Category: Other stromal tumours

Entities:

- Spindle cell gonadal stromal tumour
  Histological patterns:
    - Myoid gonadal stromal tumour
    - Fibroma/thecoma
- Stromal tumour, no specific type

Relevant differences with the current classification are presented below.

### Changes Within the Group of Stromal Tumours

1. *'Leydig cell tumour, fumarate hydratase‐deficient’ is included as a distinct entity.*

**Figure 2:**
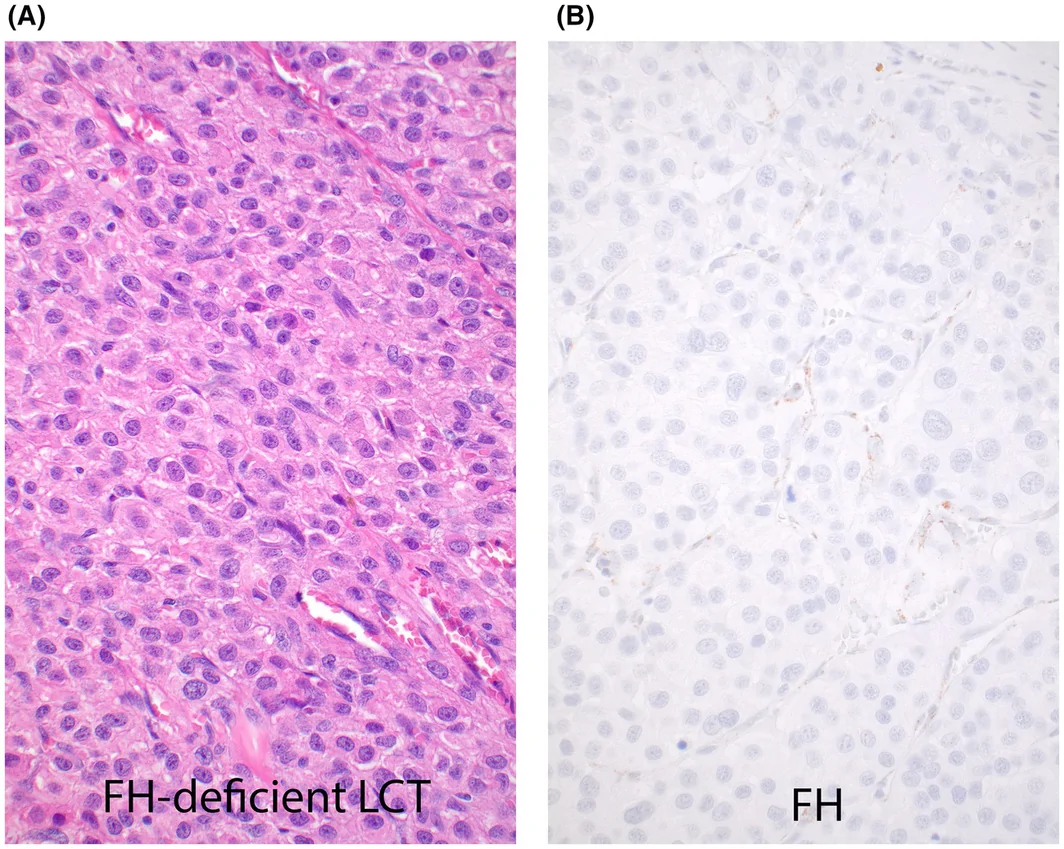
Leydig cell tumour (LCT), fumarate hydratase (FH)‐deficient (panel **A**) demonstrates loss of FH expression with immunohistochemistry (panel **B**).

Rationale: Rare Leydig cell tumours harbour pathogenic *FH* variants that result in functional loss of fumarate hydratase.^24^, ^25^ These tumours can be identified with immunohistochemistry, as they show loss of FH expression (Figure 2A,B).^2^ The first documentation of these neoplasms included a tumour affecting a patient with a known germline *FH* alteration and family history of hereditary leiomyomatosis and renal cell carcinoma.^24^ More recent studies demonstrated that *FH* alterations occurred largely in Leydig cell tumours with malignant clinical behaviour or aggressive histopathological features.^25^ In summary, these tumours differ from typical Leydig cell tumours in that: (1) they have a distinct driver; (2) they may show association with hereditary leiomyomatosis and renal cell carcinoma; and (3) most appear to exhibit aggressive clinical or pathological features. Additionally, all cases reported to date have occurred in adult patients. Therefore, we believe that these tumours represent a distinct clinicopathological entity.^26^ Moreover, renaming these tumours as ‘Leydig cell tumours, fumarate hydratase‐deficient’ highlights their association with a genetic alteration that may require germline assessment, a fact that is not widely known by clinicians.

2 *‘Spindle cell gonadal stromal tumour’ is introduced as an entity that includes ‘fibroma/thecoma’ and ‘myoid gonadal stromal tumour’ as histological patterns.*

Rationale: In the current classification (WHO 2022), fibroma/thecoma and myoid gonadal stromal tumour are considered separate entities.^1^ Although subtle histological differences have been described, both are indolent pure spindle cell neoplasms with significant overlap.^27^, ^28^, ^29^, ^30^, ^31^, ^32^, ^33^, ^34^, ^35^ By definition, myoid gonadal stromal tumour co‐expresses SMA and S100; however, these markers are largely non‐specific and known to stain many other TSCSTs.^30^, ^31^, ^32^, ^33^, ^35^, ^36^ Molecular studies have shown that TSCSTs with pure or predominant spindle cell components harbour recurrent patterns of multiple chromosomal gains suggestive of a global shift in ploidy, but alterations unique to myoid gonadal stromal tumour have not been identified.^36^, ^37^, ^38^, ^39^ In practice, distinction between fibroma/thecoma and myoid gonadal stromal tumour is difficult (and often subjective) even among genitourinary pathologists. However, this distinction is not critical since both tumours are clinically indolent, without any malignant examples reported to date, to the best of our knowledge. Given the significant clinicopathological overlap between tumours that have been reported as fibroma/thecoma and myoid gonadal stromal tumour, we believe that the introduction of an overarching entity (‘spindle cell gonadal stromal tumour’) is justified and will be beneficial for diagnostic purposes. Specifically, we think that introducing ‘spindle cell gonadal tumour’ as an entity may prevent excessive workups and expert consultations for tumours that are invariably indolent. In this classification, fibroma/thecoma and myoid gonadal stromal tumour are included as histological patterns of ‘spindle cell gonadal stromal tumour’. For instance, neoplasms with findings suggestive of myoid gonadal stromal tumour or fibroma/thecoma can be signed out as ‘spindle cell gonadal stromal tumour with features of myoid gonadal stromal tumour’ or ‘spindle cell gonadal stromal tumour with features of fibroma/thecoma’, respectively. Otherwise, those in which a clear‐cut distinction is not possible can be signed out simply as ‘spindle cell gonadal stromal tumour’.

#### Mixed sex cord‐stromal tumours group

This group encompasses neoplasms that contain a mixture of sex cord and stromal components. The latter elements include neoplastic Leydig cells or spindle cells of purported gonadal stromal origin/differentiation. The proposed classification of sex cord‐stromal tumours is presented below.

Group: sex cord‐stromal tumours

Category: Mixed sex cord‐stromal tumours.

Entities:

- Sertoli–Leydig cell tumour
- Mixed sex cord‐stromal tumour, no specific type
  Histological patterns:
    - Sertoli‐stromal cell tumour

Relevant differences with the current classification are mentioned below.

### Changes Within the Group of Sex Cord‐Stromal Tumours

1. *‘Sertoli–Leydig cell tumour’ is provisionally included as an entity.*

Although a few Sertoli–Leydig cell tumours have been reported in the testis, their existence has been debated by experts who believe that Leydig cells represent largely an entrapped non‐ neoplastic component. Recently, testicular tumours closely resembling ovarian Sertoli–Leydig cell tumours have been documented, including one with a *DICER1* RNase IIIb domain hotspot mutation.^39^, ^40^ Hence, we think that it is reasonable to include this tumour type as a provisional entity as long as the diagnosis is restricted to neoplasms with prototypical features (i.e. closely resembling true ovarian counterparts). Molecular analyses demonstrating *DICER1* alterations may provide additional support for the diagnosis, but are not required.

2 *‘Mixed sex cord‐stromal tumour, no specific type’ includes ‘Sertoli‐stromal cell tumour’ as a histological pattern.*

**Figure 3:**
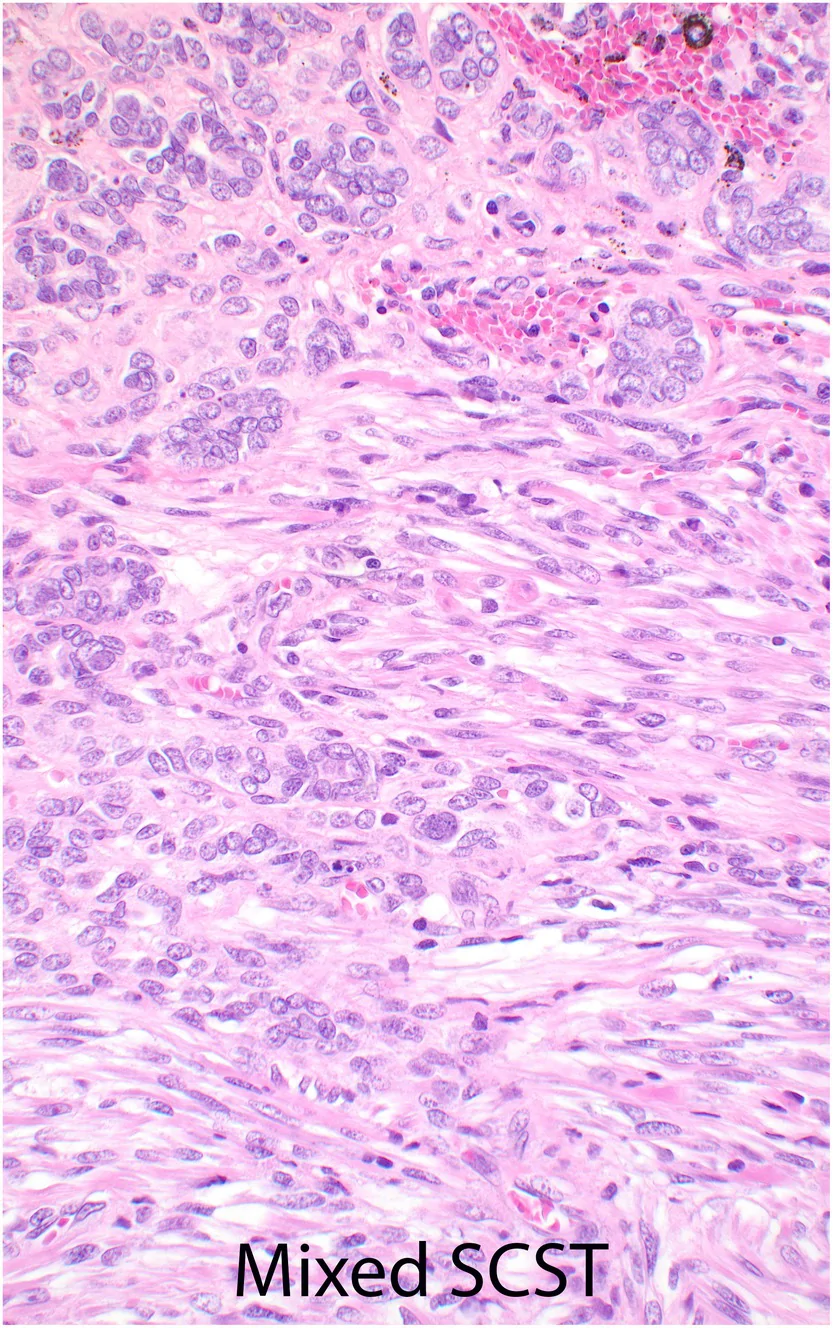
Mixed sex cord‐stromal tumour, no specific type, containing sex cord and spindle cell stromal elements. [Colour figure can be viewed at wileyonlinelibrary.com]

Mixed sex cord‐stromal tumours of no specific type include a group of neoplasms with sex cord and spindle cell stromal components (Figure 3).^41^ The sex cord elements contain primitive (i.e. immature) Sertoli‐like cells or granulosa‐like cells arranged in structures indicative of sex cord‐differentiation (e.g. nests, trabeculae, cords, ribbons, tubules). In most tumours, the sex cord components are intermingled with short fascicles and/or whorls of neoplastic spindle cells of purported gonadal stromal origin. A subset of neoplasms within this group contains germ cells that have been interpreted as an entrapped non‐ neoplastic component.^41^ However, two recent studies have suggested that they may represent a true neoplastic component akin to spermatocytic tumour.^42^, ^43^ Tumours fitting the above description have also been reported as ‘Sertoli‐stromal cell tumour’ in the literature when there is evidence of Sertoli cell differentiation with at least focal tubular architecture. Studies have shown that these neoplasms do not show unique clinicopathological or molecular features; hence, they are considered a histological pattern of mixed sex cord‐stromal tumour, usual type in this classification.^39^

## Conclusions

We present a structured hierarchical classification of TSCSTs that incorporates data from recent clinicopathological and molecular studies. This classification is based on the best evidence presently available but certainly does not intend to represent a definitive scheme since much remains to be studied in this field. However, a structured classification with a well‐defined organizational logic offers flexibility for including new entities and re‐categorizing existing ones in the future. The TESST classification of sex cord‐stromal tumours is also hierarchical, with successive tiers defined based on recognizable pathological and/or biological characteristics. The first tier, ‘group’, is defined by the compartment tumours belong to (sex cord, stroma or both). The second tier, ‘category’, is defined by the phenotype of the tumour, taking non‐neoplastic sex cord and stromal elements as a reference. The third tier, ‘entity’, refers to groups of tumours with distinct clinicopathological and/or molecular features that justify their consideration as a specific type. Finally, the fourth tier, ‘histological pattern’, refers to recurrent morphological or phenotypic variations that are not associated with unique clinicopathological or molecular features, suggesting that they represent part of the spectrum of a specific entity.

Many of the nomenclature changes and re‐categorizations introduced in this classification intend to highlight issues that are clinically relevant. For instance, the addition of the descriptor ‘benign’ to testicular juvenile granulosa cell tumour underscores that, unlike ovarian counterparts, these testicular tumours are consistently indolent and do not require aggressive treatment or long‐term follow‐up after complete resection. Similarly, the inclusion of ‘inflammatory and nested testicular sex cord tumour’ as an entity is not only justified based on the definition of ‘entity’ put forth by the classification, but it is also intended to separate these malignant tumours from Sertoli cell tumours, which are largely (~90%) indolent. A similar argument can be used to justify the distinction of Leydig cell tumour, fumarate deficient‐type from Leydig cell tumour, usual type.

This classification has limitations that must be briefly mentioned. First, given the rarity of TSCSTs, it relies on relatively limited datasets. Second, there is limited molecular validation for some entities. Third, there is still a considerable degree of interobserver variability that may have impacted existing datasets. Finally, some entities remain provisional, pending additional studies. Nonetheless, this classification incorporates the best available up‐to‐date data and represents a significant refinement of prior schemes.

In conclusion, sex cord‐stromal tumours remain relatively understudied within the field of testicular pathology. This has resulted in classifications that rely exclusively on morphology and comparisons with ovarian counterparts. We hope that this proposed new classification will provide a framework for improving categorization of these rare tumours based on the best available evidence, incorporating molecular data when available. Going forward, a more precise classification should help refine clinical management and allow future research to be performed on less heterogeneous tumour categories.

## Author contributions

Development of the classification: All authors. Manuscript draft: Andres M. Acosta. Critical manuscript revision: All authors.

## Conflict of interest

The authors declare that they do not have conflicts of interest pertaining to the contents of this manuscript.

## Funding information

The consensus activities held by the study group were funded by the Genitourinary Pathology Society (GUPS) and the International Society of Urological Pathology (ISUP).

## Consent for publication

The authors of the paper have reviewed the manuscript and provide consent for publication.

## Data Availability

Data sharing not applicable to this article as no datasets were generated or analysed during the current study.
